# Gender Differences in Predictors of Depressive Symptoms among Older Hispanic Women in the United States

**DOI:** 10.1093/geroni/igaf122.2459

**Published:** 2025-12-31

**Authors:** Jaminette Nazario-Acevedo, Takashi Yamashita

**Affiliations:** University of Maryland, Baltimore & Baltimore County, Martinsburg, West Virginia, United States; University of Maryland, Baltimore County, Baltimore, Maryland, United States

## Abstract

Older Hispanic women are more likely to experience depressive symptoms than older Hispanic men in the United States. Mental health challenges faced by older Hispanic adults, in general, and their gender differences, in particular, are often overlooked. Previous research highlights that a form of social relationship --- marriage, can serve as a protective factor for mental health. However, gender differences in specific factors associated with depressive symptoms among older Hispanic women and men have not been extensively examined. Given the intersectionality theory, the present study compared the known predictors of depressive symptoms among older Hispanic women and men. Nationally representative samples of Hispanic adults aged 51 years and older (n = 2564) are obtained from the 2018 Health and Retirement Study. Binary logistic regression shows that married older Hispanic adults (Odds ratio = 0.69, p < 0.05) are less likely to be depressed than their counterparts. No gender difference in the marital status on depressive symptoms is observed. However, there is a significant gender difference in the effect of educational attainment. Older Hispanic women with a college degree or higher (Odds ratio = 0.57, p < 0.05) are less likely to be depressed than their counterparts. Yet, educational attainment is not associated with lower depressive symptoms among men (Odd-ratio = 1.02, p > 0.05). Findings suggest differential impacts of socioeconomic status on mental health by gender. To improve the mental health of older Hispanic adults in the United States, a more targeted and gendered public health approach may be considered.

